# The Role of Intimate Partner Violence (IPV) in Mediating Between Stress and Depression Among Pregnant and Childbearing Women

**DOI:** 10.1155/nrp/8503712

**Published:** 2026-01-13

**Authors:** Ola Ali-Saleh, Ofra Halperin

**Affiliations:** ^1^ Nursing Department, Max Stern Academic College of Emek-Yezreel, Mizra, Israel

**Keywords:** childbearing women, COVID-19, intimate partner violence (IPV), mental health, pregnant women

## Abstract

This study examines depression and intimate partner violence (IPV) among Israeli women during the COVID‐19 pandemic’s second wave (which took place between June and October 2020). The participants were 240 pregnant and 310 nonpregnant women of childbearing age. No statistically significant differences were found between these groups with respect to the levels of stress, depression, and IPV. Forty percent (*n* = 220) of participants were classified within the clinical range of depression, and two‐thirds (*n* = 376) reported experiencing IPV. Muslim women reported IPV at higher rates compared to Jewish women. Factors related to an elevated risk of depression included being Muslim, having lower income, being unemployed, having higher stress, and IPV. IPV mediated the stress–depression relationship. The findings emphasize the need for accessible screening tools and targeted intervention programs, particularly for minority populations.

**Summary**

Our study examines depression and IPV among Israeli women during COVID‐19. Risk factors are as follows: Muslim faith, low income, unemployment, stress, and IPV. The IPV‐mediated stress–depression link was observed. Findings emphasize the need for screening tools and interventions, especially for minorities.

## 1. Introduction

Mental health disorders were already exerting a major burden on health systems across the globe before the COVID‐19 pandemic [1]. Depression is the most common mental disorder [2]. It is also one of the most emergent and prevalent mental health problems in the context of natural disasters [3]. The onset of the COVID‐19 pandemic was accompanied by a stark rise in depressive disorders. Across the globe, an additional 76.2 million cases of major depressive disorders (MDDs) were reported in 2020 [4]. During the COVID‐19 pandemic, symptoms of depression appeared more than three times as often as before [5, 6]. Multiple studies have shown that women are more affected by depression than men [2, 4, 7]. According to a CDC study in the United States, about one in 10 women of reproductive age reported symptoms suggesting they had recently experienced an episode of major depression [8].

Research has indicated that during disasters, such as disease outbreaks, women were found to be more susceptible to psychopathological outcomes [9, 10], including stress [11, 12], post‐traumatic stress disorder (PTSD) [13], and depression [14, 15]. In addition, when more vulnerable groups, such as pregnant women, are exposed to high levels of psychological stress, they have a greater tendency to develop mental health issues. Several studies conducted during the first wave of the COVID‐19 pandemic, in countries such as Turkey [16], Israel [17], Canada [18], and China [19], as well as worldwide reviews [20, 21], showed that pregnant women exhibit a significantly higher rate of depressive symptoms than other groups. Akgor et al. [16] survey of 297 pregnant women in Turkey during May 2020 revealed high scores on the Hospital Anxiety and Depression Scale for both anxiety and depression (7.94 ± 4.03 and 7.23 ± 3.84, respectively). Perinatal depression symptoms were assessed among Israeli women who were pregnant or up to six months postpartum, during COVID‐19, and a significantly higher prevalence was reported by Arab women than Jewish women (58% vs. 36%, respectively, *p* < 0.001) [17]. Canadian pregnant women exhibited elevated rates of anxiety (57%) and depression symptoms (37%) as a result of COVID‐19 versus nonpandemic times (10% and 25%, respectively) [18]. The impact of COVID‐19 on pregnant women’s mental health in China showed higher rates of anxiety and depressive symptoms than before the outbreak [19]. Sun et al. [20] conducted a review of 11,187 individuals and found that the prevalence of anxiety and depression among pregnant women during the COVID‐19 pandemic was higher than that of control groups. Campos‐Garzón et al. [21] reviewed 31 studies evaluating 30,049 pregnant women and indicating increased levels of anxiety and depression symptoms compared to the pre‐COVID‐19 era.

Depression during pregnancy can have an adverse effect on the mother’s physical and emotional well‐being, as well as on the fetus’ physiology and health. Among the possible repercussions of depression for the fetus are preterm labor and low birth weight [22], disrupted mother–infant bonding, increased irritability and decreased activity, delayed cognitive and language development, lower IQ, and greater prevalence of psychiatric and emotional problems [23, 24]. Depression during pregnancy can also lead to delayed neuropsychiatric development [25] and delayed emotional and linguistic development [26] in these mothers’ offspring.

The lockdowns imposed during the COVID‐19 pandemic had social and economic repercussions for family dynamics. Being forced to stay at home affected family income, interpersonal bonds, and the mental and emotional well‐being of all family members [4, 27], but research shows that women are more likely to be affected by the social and economic consequences of the pandemic [28, 29]. The lockdowns also caused women to be more exposed to abusive partners and known risk factors, while at the same time limiting their access to social services. Indeed, according to the World Health Organization (WHO) [30], the COVID‐19 pandemic led to considerable concern regarding the escalation of intimate partner violence (IPV) and its negative consequences.

According to various studies taken around the world, incidents of domestic violence increased in response to stay‐at‐home orders [31]. Close to one‐third (27%) of all women of childbearing age (aged 15–49 years) across the globe reported having experienced IPV at least once in their lifetime [30, 32]. A study conducted by the WHO, which examined women’s health and domestic violence in several countries, found that between 15% and 71% of women who had ever been in a relationship experienced physical and/or sexual violence from a current or former partner at some stage in their lives [33]. During the lengthy lockdowns mandated by the COVID‐19 pandemic, IPV has only intensified [34].

IPV is considered a major public health and human rights issue [35]. Among its wide‐ranging health consequences are fear, injuries, chronic physical and mental health problems, and premature death [30]. The psychological consequences of IPV include depression, anxiety, PTSD, and suicidal thoughts [36]. During pregnancy, IPV is often expressed as economic, physical, or verbal abuse and has been associated with stress and depression among pregnant women [37, 38].

Halperin and her colleagues [39] found that in Israel, a high percentage of women of childbearing age reported IPV, perceived stress, COVID‐19‐related stress, and depression. In particular, IPV mediates a positively proportional relationship between the stress caused by COVID‐19 and depression, which subsequently influenced depression (i.e., greater stress was related to more IPV). Domestic and homebound violence explained the association between stress and depression. The increased prevalence of MDD and anxiety disorders in 2020 was associated with elevated rates of SARS‐CoV‐2 infection and decreased human mobility [4]. Women are more likely to be survivors of domestic violence, the prevalence of which increased during periods of lockdown and stay‐at‐home orders [31, 40].

In Israel, the second wave of the COVID‐19 pandemic (which occurred between June 2020 and October 2020) reached its peak in September 2020, when several thousand new cases were confirmed daily, representing the highest incidence rate per million citizens worldwide, with approximately 9000 new infections registered in one day. In response, the government introduced a new lockdown starting on September 18, 2020 [41].

According to the Israel Central Bureau of Statistics (ICBSI) [42], there are 9,842,000 citizens in Israel, and most of them are Jews (73.2%), with a large (21%) Arab minority. Members of the Arab minority are mostly Muslim (83.3%), with significantly smaller Christian (7.7%) and Druze (9%) groups [43].

The Arab minority in Israel experiences higher rates of mental disorders than the Jewish population [44]. A study conducted in Israel, which examined the levels of mental distress, found that only 30% of the Arab participants did not report symptoms of mental distress, as compared to 75% in Jewish society [45]. Studies conducted during the COVID‐19 pandemic in Israel found that approximately 41% of Arab women were classified in the clinical range of depression [39]. Taubman‐Ben‐Ari and her colleagues [46] demonstrated that Arab pregnant women were more anxious than their Jewish counterparts during the COVID‐19 pandemic. The rate of postpartum depression was on average 10.3% among the study groups, being significantly more prevalent in Arab women than Jewish women (20.8% vs. 7%, respectively) [47]. Arab women are more vulnerable to mental health issues and are subject to continuous stress, which affects their physical and mental health [44]. Similarly, international studies have found higher rates of anxiety among women from minority populations as compared to other groups [48, 49].

Therefore, the aim of this study was to investigate the prevalence and predictors of depression and IPV among Israeli women during the second wave of the COVID‐19 pandemic and the relationship between them. In addition, the study aimed to examine the differences in the prevalence of depression and violence against women among pregnant women and women of childbearing age in reference to the cultural differences between them. To this end, we proposed a mediation model to assess the relationship between IPV and such mental health variables as depression and stress among pregnant and childbearing women during the second wave of the COVID‐19 pandemic. Very few studies have examined mental health issues of pregnant and childbearing age women regarding IPV during the second wave of the pandemic.

## 2. Materials and Methods

This is a cross‐sectional study of pregnant women and women of childbearing age (excluding postnatal women) among Jewish and Arab Israelis. The women were recruited through existing forums on social networks and completed an online self‐report questionnaire during the second wave of the COVID‐19 pandemic. The study used a snowball sample method by asking the participants to forward the questionnaire link to other relevant individuals. Participation in this study was voluntary; incentives were not offered. Inclusion criteria were Israeli women from 18 to 45 years of age who were married or in a relationship. The questionnaire included demographic variables and perceived stress scale (PSS), IPV, and depression (Patient Health Questionnaire‐2 [PHQ‐2]). The Ethics Committee of the Yezreel Valley College approved the study (Reference Number: 91‐2020 YVC EMEK).

### 2.1. Participants’ Demographic Variables

The demographic variables recorded in this study included age, number of children, religion and religiosity, level of education, income, employment, and type of residence. Participants in this study were 550 women: 240 pregnant women (43.6%) and 310 nonpregnant women of childbearing age (56.4%). Most women were 25–34 years old (64%, *n* = 352), and others were mostly 35–45 years old (29%, *n* = 161), with the pregnant women being significantly younger than the nonpregnant ones (Table 1). The participants had up to five children, with a mean of 1.65 children (SD = 1.25). The pregnant women had significantly fewer children than the nonpregnant ones. The women were Muslim (45%, *n* = 249), Christian (35%, *n* = 191), and Jewish (20%, *n* = 110), with no differences regarding the number of children of a given woman of any faith. Approximately 80% (*n* = 443) of the participants had an academic education, and approximately 78% (*n* = 431) were working outside the home. The reported levels of income of participating women were as follows: ∼45% (*n* = 249) below average, ∼33% (*n* = 181) around average, and ∼22% (*n* = 120) above average. Approximately 57% (*n* = 313) of the women were urban, 26% (*n* = 142) were secular, 43% (*n* = 236) were partly religious, and 31% (*n* = 172) were religious. No meaningful group differences were found for most of the sociodemographic characteristics, except for age and number of children.

**Table 1:** Participants’ sociodemographic characteristics (*N* = 550).

Characteristics	Total sample *N* (%)	Pregnant women *N* (%)	Nonpregnant women of childbearing age *N* (%)	Difference
Age group, years				
18–24	35 (6.4)	30 (12.5)	5 (1.6)	*χ* ^2^ (2) = 59.21 *p* < 0.001
25–34	354 (64.4)	174 (72.5)	180 (58.1)	
35–45	161 (29.3)	36 (15.0)	125 (40.3)	
Mean number of children (SD), range	1.65 (1.25), 0–5	1.24 (1.20), 0–5	1.96 (1.20), 0–5	*t* (548) = 6.97, *p* < 0.001
Religion				
Jewish	110 (20.0)	48 (20.0)	62 (20.0)	*χ* ^2^ (2) = 5.73 *p* = 0.057
Muslim	249 (45.3)	121 (50.4)	128 (41.3)	
Christian	191 (34.7)	71 (29.6)	120 (38.7)	
Education (%)				
Less than academic	107 (19.5)	48 (20.0)	59 (19.0)	*Z* = 0.28 *p* = 0.776
Academic	443 (80.5)	192 (80.0)	251 (81.0)	
Employment status				
Homemaker	69 (12.5)	39 (16.3)	30 (9.7)	*χ* ^2^ (3) = 6.21 *p* = 0.102
Salaried	376 (68.4)	161 (67.1)	215 (69.4)	
Freelance	37 (6.7)	15 (6.3)	22 (7.1)	
Other	68 (12.4)	25 (10.4)	43 (13.9)	
Works outside the home				
Yes	431 (78.4)	178 (74.2)	253 (81.6)	*Z* = 2.10 *p* = 0.035
Income^a^				
Much below average	105 (19.1)	50 (20.8)	55 (17.7)	*χ* ^2^ (3) = 4.63 *p* = 0.201
Somewhat below average	144 (26.2)	71 (29.6)	73 (23.6)	
Average	181 (32.9)	71 (29.6)	110 (35.5)	
Somewhat above average	120 (21.8)	48 (20.0)	72 (23.2)	
Residence				
Urban	313 (56.9)	128 (53.3)	185 (59.7)	*Z* = 1.49 *p* = 0.136
Rural	237 (43.1)	112 (46.7)	125 (40.3)	
Religiosity				
Secular	142 (25.8)	54 (22.5)	88 (28.4)	*χ* ^2^ (2) = 4.22 *p* = 0.121
Partly religious	236 (42.9)	101 (42.1)	135 (43.5)	
Religious	172 (31.3)	85 (35.4)	87 (28.1)	

^a^The question on the questionnaire was as follows: The average gross salary for an employee is about 10,500 NIS per month, and for a family, it is about 18,000 NIS gross per month. What is your income level compared to the average income?

### 2.2. Instruments

#### 2.2.1. Stress

The PSS developed by Cohen and Williamson was used [50]. The scale measures perceptions and feelings related to general stress levels in recent times using grades from 0 = never to 4 = often. The range of possible scores is between 0 and 40, where higher scores indicate higher levels of perceived stress. Scores between 0 and 13 were considered as a low stress level, scores between 14 and 26 were considered as a moderate stress level, and scores ranging between 27 and 40 were considered as a high stress level. The internal consistency (Cronbach’s *α*) for the full ten‐item scale was 0.86.

#### 2.2.2. Depression

The PHQ‐2 developed by Kroenke and his colleagues [51] was used. This study’s questionnaire consists of the first two items of the PHQ‐9. Both items measure a depressed mood and loss of interest (anhedonia), of which at least one item is required to be considered positive: suspected major depression (MDD) or any depressive disorder according to the DSM‐IV. PHQ‐2 scores can range from 0 to 6, and a cutoff point of ≥ 3 indicates significant clinical depression. The correlation between the two items in this was *r* = 0.74 (*p* < 0.001).

#### 2.2.3. Questionnaire on IPV

IPV (violence perpetrated by the participant’s spouse) was assessed using a questionnaire consisting of 10 items that could be graded as one of the following options: (1) never, (2) rarely, (3) often, and (4) always. The questions were those used by the Israeli Ministry of Health, based on the IPV U.S. Prevention Services Task Force [52], as well as on research conducted in Israel [37]. A woman was defined as a victim of violence if she answered affirmatively to at least one of the 10 questions. Cronbach’s α of the entire scale was 0.82. Types of violence were defined as physical or sexual violence: Items 6, 9, and 10 (Cronbach’s *α* = 0.72); emotional or verbal violence: Items 1, 2, 7, and 8 (Cronbach’s *α* = 0.56); and social or economic violence: Items 3, 4, and 5 (Cronbach’s *α* = 0.67). Total scores were computed with item means.

The questionnaires were originally written in English and translated in two stages: first from English into Hebrew and Arabic and then back‐translated by other translators from Hebrew and Arabic to English. Data collection was completed during the second lockdown period, September 18 to October 17, 2020, corresponding to the lockdown imposed by the Ministry of Health to contain the COVID‐19 pandemic [53].

### 2.3. Data Analysis

The data were analyzed using IBM SPSS Statistics (Version 29). Depression was dichotomized with a cutoff point of ≥ 3 signifying the clinical range of depression. IPV was defined as existing if the woman agreed with at least one of the relevant questions. To assess the associations between demographic variables and depression/IPV, (yes/no) odds ratios (OR) and 95% confidence intervals (CIs) were calculated. The measures of violence deviated from a normal distribution (skewness = 1.68 to 4.46; SE = 0.10) and were thus log transformed. Group differences in the study variables were assessed with *t*‐tests, and Pearson’s correlations were calculated between the study variables. Multiple hierarchical logistic regressions were calculated for the dichotomous categorization of depression as the dependent variable. Group and demographic/socioeconomic variables were entered in the first step; the independent variable of stress was entered second; and the mediating variables of IPV and its dimensions were entered in the third step. To evaluate the mediating role of IPV in the association between stress and depression, the process procedure [54] was used. This procedure is added to the SPSS software and is used to assess mediation, moderation, and conditional processes. It is based on regression analyses and offers bootstrapping, analysis of indirect effects, simple slope analysis, and graphical presentation of the results. In this study, Model 4 for a binary dependent variable was used, with 5000 bootstrap samples and 95% CI. A *p*‐value of *p* < 0.05 was considered to be the threshold of statistical significance.

## 3. Results

### 3.1. Descriptive Results

Of the 550 women, 220 (40.0%) were classified within the clinical range of depression, and approximately two‐thirds (*N* = 376, 68.4%) reported some IPV. Approximately a third of the women (*N* = 185, 33.6%) were survivors of IPV and were classified in the clinical range of depression. Demographic and socioeconomic associations with the categorizations of depression and IPV were examined with logistic regressions (see Table 2). Age group was controlled for due to the significant differences shown in Table 1 (defined as a dummy variable 0 = 18–34 years and 1 = 35–45 years). As the age group was significantly associated with the number of children (*r* = 0.49 and *p* < 0.001), the control for age accounted for these group differences as well.

**Table 2:** Depression and IPV by sociodemographic variables and PSS (*N* = 550).

Predictors	Depression (*N* = 550)				Any type of violence (*N* = 550)			
	Yes (*n* = 220) *n* (%)	No (*n* = 330) *n* (%)	*p*	OR (95% CI)	Yes (*n* = 376) *n* (%)	No (*n* = 174) *n* (%)	*p*	OR (95% CI)
Group			0.243				0.805	
Pregnant	101 (42.1)	139 (57.9)		0.81 (0.56–1.16)	161 (67.1)	79 (32.9)		1.05 (0.72–1.53)
Nonpregnant childbearing women	119 (38.4)	191 (61.6)		1.00 (reference)	215 (69.4)	95 (30.6)		1.00 (reference)
Age group			0.104				0.109	
18–24	19 (54.3)	16 (45.7)		1.58 (0.76–3.30)	28 (80.0)	7 (20.0)		1.55 (0.63–3.80)
25–34	132 (37.3)	222 (62.7)		0.79 (0.54–1.16)	232 (65.5)	122 (34.5)		0.74 (0.49–1.11)
35–44	69 (42.9)	92 (57.1)		1.00 (reference)	116 (72.0)	45 (28.0)		1.00 (reference)
Religion			< 0.001				0.008	
Jewish	38 (34.5)	72 (65.5)		1.00 (reference)	66 (60.0)	44 (40.0)		1.00 (reference)
Muslim	129 (51.8)	120 (48.2)		2.03 (1.28–3.24)	187 (75.1)	62 (24.9)		2.00 (1.24–3.23)
Christian	53 (27.7)	138 (72.3)		0.73 (0.44–1.21)	123 (64.4)	68 (35.6)		1.22 (0.75–1.99)
Religiosity			< 0.001				0.011	
Secular	38 (26.8)	104 (73.2)		1.00 (reference)	83 (58.5)	59 (41.5)		1.00 (reference)
Partly religious	97 (41.1)	139 (58.9)		1.93 (1.22–3.04)	171 (72.5)	65 (27.5)		1.90 (1.22–2.95)
Religious	85 (49.4)	87 (50.6)		2.69 (1.67–4.33)	122 (70.9)	50 (29.1)		1.75 (1.09–2.79)
Education			0.340				0.038	
Nonacademic	47 (43.9)	60 (56.1)		1.23 (0.80–1.89)	82 (76.6)	25 (23.4)		1.68 (1.03–2.74)
Academic	173 (39.1)	270 (60.9)		1.00 (reference)	294 (66.4)	149 (33.6)		1.00 (reference)
Income level			< 0.001				0.002	
< Average	120 (48.2)	129 (51.8)		1.90 (1.34–2.69)	187 (75.1)	62 (24.9)		1.82 (1.26–2.65)
≥ Average	100 (33.2)	201 (66.8)		1.00 (reference)	189 (62.8)	112 (37.2)		1.00 (reference)
Residence			0.393				0.922	
City	120 (38.3)	193 (61.7)		0.86 (0.61–1.21)	213 (68.1)	100 (31.9)		0.98 (0.68–1.41)
Other	100 (42.2)	137 (57.8)		1.00 (reference)	163 (68.8)	74 (31.2)		1.00 (reference)
Work outside home			0.013				0.174	
Yes	161 (37.4)	270 (62.6)		1.00 (reference)	289 (67.1)	142 (32.9)		1.00 (reference)
No	59 (49.6)	60 (50.4)		1.68 (1.12–2.54)	87 (73.1)	32 (26.9)		1.37 (0.87–2.16)
Number of children			0.576				< 0.001	
0	44 (36.1)	78 (63.9)		1.00 (reference)	67 (54.9)	55 (45.1)		1.00 (reference)
1	51 (38.1)	83 (61.9)		1.09 (0.65–1.81)	88 (65.7)	46 (34.3)		1.57 (0.95–2.60)
2–6	125 (42.5)	169 (57.5)		1.27 (0.80–2.04)	221 (75.2)	73 (24.8)		2.65 (1.62–4.32)
PSS stress (mean ± SD, 95% CI)	23.64 ± 6.16 (22.82–24.45)	15.95 ± 6.13 (15.28–16.61)	< 0.001	1.23 (1.18–1.28)	20.36 ± 6.91 (19.66–21.06)	16.14 ± 6.99 (15.09–17.18)	< 0.001	1.09 (1.06–1.12)

Significant associations with depression and IPV were found. Both depression and IPV were significantly more common among Muslim women than among Jewish women. They were also more common among religious and partly religious women than among secular women. Depression was more common among women reporting a below‐average income and not working outside the home, than among women reporting an average or above‐average income and working outside the home. IPV was more common among women with a nonacademic education and caring for at least two children than among women with an academic education and caring for one child or none. Finally, stress was associated with both depression and IPV, such that higher levels of stress increased the odds for being classified in the clinical range of depression, as well as for reporting the existence of IPV.

Approximately half of the women who reported IPV were classified in the clinical range of depression (*N* = 185, 49.2%), as compared with about one‐fifth of the women who did not report IPV (*N* = 35, 20.1%), a significant difference (*p* < 0.001). Likewise, approximately half to two‐thirds of the women who reported physical/sexual violence, emotional/verbal violence, or social/economic violence were classified in the clinical range of depression, as compared with approximately a quarter to a third of the women who did not report IPV, all significant differences (see Table 3).

**Table 3:** The relationship between IPV and depression (*N* = 550).

	Depression		*p*	OR (95% CI)
	Yes (*n* = 220) *n* (%)	No (*n* = 330) *n* (%)		
Any violence				
Yes (*n* = 376, 68.4%)	185 (49.2)	191 (50.8)	< 0.001	3.83 (2.51–5.84)
No (*n* = 174, 31.6%)	35 (20.1)	139 (79.9)		
Physical/sexual violence				
Yes (*n* = 93, 16.9%)	62 (66.7)	31 (33.3)	< 0.001	3.76 (2.35–6.04)
No (*n* = 457, 83.1%)	158 (34.6)	299 (65.4)		
Emotional/verbal violence				
Yes (*n* = 308, 56.0%)	160 (51.9)	148 (48.1)	< 0.001	3.27 (2.26–4.73)
No (*n* = 242, 44.0%)	60 (24.8)	182 (75.2)		
Social/economic violence				
Yes (*n* = 223, 40.5%)	113 (50.7)	110 (49.3)	< 0.001	2.10 (1.48–2.98)
No (*n* = 327, 59.5%)	107 (32.7)	220 (67.3)		

Means in Table 4 reveal moderate stress levels, low IPV levels (the continuous score), and approximately 40% of the women who were classified as suffering from depression. No significant group differences were detected, controlling for age group. All possible correlations among any pair of variables were found to be positive and significant. That is, higher stress was positively associated with IPV and depression, and higher IPV was associated with higher odds of being classified in the clinical range of depression.

**Table 4:** Means and standard deviations by group and correlations for the study variables (*N* = 550).

	**Total sample** ***M* (SD)**	**Pregnant women** ***M* (SD)**	**Nonpregnant women of childbearing age** ***M* (SD)**	**Group difference** **p**	**1.**	**2.**	**3.**	**4.**	**5.**	**6.**

1. PSS stress	19.02 (7.20)	19.33 (7.47)	18.78 (6.99)	0.418	1					
2. Physical/sexual violence	0.12 (0.36)	0.12 (0.32)	0.12 (0.38)	0.579	0.22^∗∗∗^	1				
3. Emotional/verbal violence	0.35 (0.45)	0.32 (0.43)	0.37 (0.47)	0.450	0.33^∗∗∗^	0.55^∗∗∗^	1			
4. Social/economic violence	0.32 (0.56)	0.32 (0.56)	0.32 (0.55)	0.543	0.24^∗∗∗^	0.55^∗∗∗^	0.56^∗∗∗^	1		
5. Total violence	0.27 (0.39)	0.26 (0.38)	0.28 (0.41)	0.998	0.33^∗∗∗^	0.75^∗∗∗^	0.89^∗∗∗^	0.85^∗∗∗^	1	
6. Depression (yes/no)	0.40 (0.49)	0.42 (0.49)	0.38 (0.49)	0.245	0.52^∗∗∗^	0.21^∗∗∗^	0.35^∗∗∗^	0.24^∗∗∗^	0.34^∗∗∗^	1

*Note:* Range: PSS stress, 0–40; violence, 0–3; depression, 0 or 1.

^∗∗∗^ *p* < 0.001.

### 3.2. Logistic Regression for Depression

As shown in Table 2, Muslim women, nonsecular women, women with lower income, and women not working outside the home had higher odds of being classified in the clinical range of depression, as compared to other women. Thus, these demographic and socioeconomic variables, as well as age group, were controlled for in further analyses (religion: 1—Muslim and 0—Jewish and Christian; level of religiosity: 1—religious and partly religious and 0—secular; income: 1—average and above average and 0—lower than average; work outside the home: 1—yes and 0—no; age group: 1—age 35–45 and 0—age 18–34). A multiple hierarchical logistic regression was calculated for depression as the dependent variable (yes/no) (see Table 5). Group (pregnant women/nonpregnant women of childbearing age) and the demographic and socioeconomic variables were entered in the first step; stress was entered in the second step; and IPV was entered in the third step. Results show that the odds for being classified in the clinical range for depression were higher for Muslim women, women with a lower‐than‐average income, and women with higher stress and higher IPV levels.

**Table 5:** Multiple hierarchical logistic regression for depression (*N* = 550).

	*B* (SE)	**p**	OR (95% CI)
Step 1			
Group (pregnant women)	0.06 (0.19)	0.763	1.06 (0.73, 1.55)
Age (older)	0.22 (0.21)	0.300	1.24 (0.83, 1.87)
Religion (Muslim)	0.70 (0.19)	< 0.001	2.01 (1.37, 2.93)
Level of religiosity (nonsecular)	0.44 (0.23)	0.060	1.55 (0.98, 2.43)
Income (average and above)	−0.51 (0.18)	0.006	0.60 (0.42, 0.87)
Work outside the home (yes)	−0.26 (0.22)	0.245	0.77 (0.50, 1.19)
Step 2			
Group (pregnant women)	−0.08 (0.23)	0.727	0.92 (0.59, 1.45)
Age (older)	0.28 (0.24)	0.253	1.32 (0.82, 2.12)
Religion (Muslim)	0.82 (0.23)	< 0.001	2.28 (1.44, 3.61)
Level of religiosity (nonsecular)	0.52 (0.27)	0.051	1.69 (0.99, 2.85)
Income (average and above)	−0.54 (0.22)	0.015	0.58 (0.38, 0.90)
Work outside the home (yes)	−0.16 (0.26)	0.535	0.85 (0.51, 1.42)
PSS stress	0.22 (0.02)	< 0.001	1.24 (1.19, 1.29)
Step 3			
Group (pregnant women)	−0.03 (0.23)	0.907	0.97 (0.62, 1.53)
Age (older)	0.19 (0.25)	0.451	1.20 (0.74, 1.95)
Religion (Muslim)	0.75 (0.24)	0.002	2.12 (1.33, 3.38)
Level of religiosity (nonsecular)	0.43 (0.27)	0.112	1.54 (0.90, 2.62)
Income (average and above)	−0.45 (0.23)	0.045	0.63 (0.41, 0.99)
Work outside the home (yes)	−0.10 (0.27)	0.710	0.90 (0.53, 1.53)
PSS stress	0.21 (0.02)	< 0.001	1.23 (1.18, 1.28)
IPV total	1.20 (0.50)	0.003	4.46 (1.68, 11.88)

*Note:* *χ* ^2^ (8) = 224.28, *p* < 0.001, Nagelkerke’s *R* ^2^ = 0.45.

In addition to the logistic regression analysis with the total score for IPV, another model was attempted with the three dimensions: physical violence, emotional violence, and social violence. However, due to relatively high VIF values (up to 1.80), and the rather high correlations between the three dimensions of IPV (*r* = 0.55 to *r* = 0.56; *p* < 0.001), each dimension was examined in a separate logistic model. Emotional/verbal violence was positively associated with the odds for classification in the clinical range of depression (*B* = 1.53; SE = 0.41; *p* < 0.001; OR = 4.60; 95% CI = 2.06, 10.27), yet the associations for physical/sexual violence and social/economic violence were not significant (physical/sexual violence: *B* = 0.74, SE = 0.52, *p* = 0.158, OR = 2.09, and 95% CI = 0.75, 5.85; social/economic violence: *B* = 0.52, SE = 0.35, *p* = 0.140, OR = 1.68, and 95% CI = 0.84, 3.35). That is, higher levels of emotional/verbal violence were associated with increased odds for classification in the clinical range of depression.

### 3.3. IPV as a Mediator

Finally, an attempt was made to assess the mediating role of IPV in the association between stress and depression. Due to the results of the logistic regressions described above, this was attempted for the total score of IPV and emotional/verbal violence (as the associations between physical/sexual violence and social/economic violence and depression were not significant). The process procedure [54] was used, employing Model 4 for a binary dependent variable, with 5000 bootstrap samples and 95% CI (Table 6). Results reveal that total IPV and emotional/verbal violence mediate the association between stress and the classification of depression within the clinical range. Higher stress was associated with higher IPV and higher emotional/verbal violence, which, in turn, were associated with higher odds for being classified in the clinical range of depression.

**Table 6:** Path coefficients and indirect effects for total and emotional/verbal IPV as mediators between stress and depression (*N* = 550).

Dependent variable (DV)	Variable	Path coefficients		Indirect effects	
		To DV estimate (SE)	To mediator estimate (SE)	Estimate (SE)	95% CI
Depression	PSS stress	1.46 (0.15) (*p* < 0.001)	0.32 (0.04) (*p* < 0.001)	0.11 (0.05)	0.03, 0.21
	IPV total	0.35 (0.12) (*p* = 0.003)			

Depression	PSS stress	1.44 (0.15) (*p* < 0.001)	0.32 (0.04) (*p* < 0.001)	0.14 (0.04)	0.07, 0.23
	IPV emotional/verbal	0.43 (0.11) (*p* < 0.001)			

## 4. Discussion

The results of the present study show that during the COVID‐19 pandemic no differences were found in stress, depression, and IPV between pregnant women and nonpregnant women of childbearing age. This finding is consistent with the results of other studies showing that women of reproductive age are vulnerable during crises, without a significant difference between pregnant and nonpregnant women [55, 56].

This study found that during the COVID‐19 pandemic, a high percentage of women (40.0%) were classified within the clinical range of depression. This finding corresponds with previous studies conducted during the first wave of the pandemic, where women exhibited heightened psychological distress manifested as depression and stress [57, 58]. Wang et al.’s [57] survey of the general population in China concerning the first wave of the COVID‐19 pandemic impact on anxiety, depression, and stress found that women reported higher levels of these psychological components than men. The general population in China, both men and women, experienced psychological distress [58]. The explanation for this could be that during the second wave the number of confirmed cases in Israel was particularly high, and a positive association emerged between the number of newly confirmed COVID‐19 cases and depressive symptoms [21]. The high level of mental distress may also have been caused by the prolonged lockdown periods and strict curfews imposed by some countries in an effort to contain the pandemic [59, 60]. The impact of COVID‐19 incorporated the combined effects of the spread of the virus, lockdowns, stay‐at‐home orders, reduced public transportation, school and business closings, and decreased social interactions [4]. As a consequence, the high prevalence of women classified with depression during the pandemic is expected.

Two‐thirds of the women in this study reported some IPV. This figure is higher than the IPV statistics published by the WHO (27%) [30]. According to the Knesset Research and Information Center [61], IPV in Israel increased during the COVID‐19 pandemic as compared to the previous year. This finding suggests that crisis conditions may motivate outbursts of partner violence toward women. Evidence from a systematic review and meta‐analysis found that most study estimates were indicative of an increase in domestic violence postlockdowns [31]. Similarly, Abujilban and her colleagues [34] compared the prevalence of IPV toward pregnant women in Jordan before and during the COVID‐19 quarantine and found increased IPV during lockdowns/quarantines. Standish and Weil [62] also examined extreme violence against women during COVID‐19, discovering that several countries reported increased rates of femicide during the pandemic, along with a consequent rise in domestic violence.

In addition, this study reported that IPV was higher among Muslim women than among Jewish women, a finding similar to the results of Daoud and her colleagues [37], who investigated IPV among Arab and Jewish women in Israel before COVID‐19. The results of the current study show that Muslim women, women with lower incomes, women who do not work outside the home, and women with higher levels of stress and IPV are more likely to be classified in the clinical range of depression than other women.

Previous studies also found that women from ethnic minorities and low‐income groups experience higher rates of mental health problems, including psychological distress [45, 63], and have higher rates of depression than women from ethnic majorities [64, 65]. Previous studies found an association between low income and IPV [37]. Women are more likely to be financially disadvantaged during the pandemic due to lower salaries, less savings, and less secure employment than their male counterparts [28, 29].

Arab society in Israel ranks lower than Jewish society on almost every socioeconomic index. National Insurance Institute data [66] reported that 45% of Arab families in Israel live below the poverty line, as do 57.8% of Arab children and 55.9% of elderly Arabs. In comparison, in 2018, the average poverty rate was 18% for the general Israeli population and 30% for children. Alfayumi‐Zeadna and her colleagues [17] examined perinatal depression symptoms during COVID‐19 in a nationwide study of Jewish and Arab women in Israel and found that a higher prevalence of perinatal depression was related to high stress levels among Arab women. Living in poverty is a known risk factor for mental distress, as it exposes people to negative life events such as chronic stress, parental distress, feelings of instability, and problems with family functioning.

It is true that Arab women are now more educated than before and participate more in the workforce and their economic independence is expanding and strengthening, yet it is still accepted that women are the main caregivers of the household and other family members. The result is that women are more exposed to the mental pressures arising from the conflicting demands of family and employment [67]. Additional career and household responsibilities due to school closures or family members becoming unwell are more likely to burden women [29].

The lockdowns during the COVID‐19 pandemic added pressure to domestic relationships, exacerbating partner violence. Women consequently experienced elevated rates of anxiety, stress, and depression [17, 68]. In Israel, the COVID‐19 pandemic caused an over twofold increase in the rates of perinatal depression symptoms among women [17]. Rahimi et al.’s [68] review of studies reporting the prevalence of anxiety among pregnant women during COVID‐19 around the world revealed that in Asian countries it ranged between 3.8% and 17.5%, whereas in Western countries it ranged between 23.9% and 72%. Research has found an association between increased IPV as a risk factor for depression and the stress experienced by postpartum women [39], as well as IPV being a significant factor associated with depression [38, 69, 70]. Shwartz et al.’s [38] research on postpartum women suffering depression in Israel found that it was linked to IPV. Chandan et al. [69] showed that in a U.K. women population those exposed to IPV had a higher incidence rate of mental illness than those not exposed to IPV. For example, anxiety and depression were associated with exposure to IPV. A study by Refaeli et al. [70] discovered that among Israeli women victims of IPV, living in shelters, depression was slightly attributed to the frequency of violence. These studies support our finding that IPV mediates the association between stress and depression. All possible pairwise correlations between stress, IPV, and depression were found to be clearly positive. Consequently, preventing violence may help reduce stress‐related depression.

Several limitations to this study should be noted. The questionnaire was completed in a manner that did not consider the women’s conditions before the survey (e.g., stress, depression, or IPV) or pregnancy follow‐up. Moreover, participation was limited to women capable of using the Internet. Another limitation of the study is the snowball sampling methodology because it might not be representative of the broader population; rather, its use may introduce bias to the data by relying on participants forwarding the questionnaire to other potentially relevant participants. In addition, the cross‐sectional design of this study may be a limitation because the participants were not followed over time; rather, they completed the questionnaire only once; hence, a cause‐and‐effect relationship cannot be established because exposure to violence and the mental health outcome issues were measured simultaneously.

## 5. Conclusion

This study found that there were no significant differences in the prevalence of stress, depression, and IPV between pregnant women and nonpregnant women of childbearing age. The above finding underlines the vulnerability of all women during times of crisis. It is also important to note that minority populations are exposed during times of crisis to more pressure, depression, and IPV.

Regular health checkups should be offered to women of childbearing age because they are particularly vulnerable to IPV and mental disorders; thus, they should be regularly monitored via health checkups. It is important to make screening tools and assessments for IPV, stress, and depression more available in diverse community, clinical, and health settings.

These findings can also be used to design intervention programs to reduce IPV and encourage a mental health intervention strategy as a follow‐up to COVID‐19, with particular emphasis on minority populations.

## Ethics Statement

The study was approved by the Max Stern Yezreel Valley College Ethical Committee, No. 91‐2020.

## Conflicts of Interest

The authors declare no conflicts of interest.

## Author Contributions

O.A‐S.’s role in the publication: initiation of the study, contribution to the conceptualization of the study, development of the research design and methods, data curation and resources, data analysis, review of the literature, writing of the initial draft of the manuscript, paper revision (during the publication process), and approval of the final version of the manuscript.

O.H.’s role in the publication: contribution to the development of the research design and methods, data analysis, review of the literature, writing of the initial draft of the manuscript, and approval of the final version of the manuscript.

## Funding

This research did not receive any specific grant from funding agencies in the public, commercial, or not‐for‐profit sectors.

## Data Availability

The data that support the findings of this study are available from the corresponding author upon reasonable request.
